# Association of *tcdA+/tcdB+ Clostridium* difficile Genotype with Emergence of Multidrug-Resistant Strains Conferring Metronidazole Resistant Phenotype

**DOI:** 10.7508/ibj.2015.03.003

**Published:** 2015-07

**Authors:** Farahnaz-Sadat Shayganmehr, Masoud Alebouyeh, Masoumeh Azimirad, Mohammad Mehdi Aslani, Mohammad Reza Zali

**Affiliations:** 1 *Foodborne and Waterborne Diseases Research Center, Research Institute for Gastroenterology and Liver Diseases, Shahid Beheshti University of Medical Sciences, Tehran, Iran; *; 2 *Dept. of Microbiology, Science and Research Branch, Islamic Azad University, Fars, Iran; *; 3 *Gastroenterology and Liver Diseases Research Center, Research Institute for Gastroenterology and Liver Diseases, Shahid Beheshti University of Medical Sciences, Tehran, Iran; *; 4 *Dept. of Microbiology, Pasteur Institute of Iran, Tehran, Iran*

**Keywords:** Multidrug resistance, *Clostridium difficile*, Metronidazole

## Abstract

**Background:**

Reduced susceptibility of *Clostridium difficile* to antibiotics is problematic in clinical settings. There is new evidence indicating the cotransfer of toxin-encoding genes and conjugative transposons encoding resistance to antibiotics among different *C. difficile* strains. To analyze this association, in the current study, we evaluated the frequency of toxigenic *C. difficile* among the strains with different multidrug-resistant (MDR) profiles in Iran.

**Methods:**

Antimicrobial susceptibility patterns and minimal inhibitory concentrations (MIC) of the isolates were determined against metronidazole, imipenem, ceftazidime, amikacin, and ciprofloxacin by agar dilution method. The association of the resistance profiles and toxigenicity of the strains were studied by PCR targeting *tcdA* and *tcdB* genes.

**Results:**

Among 86 characterized strains, the highest and lowest resistance rates were related to ciprofloxacin (97%) and metronidazole (5%), respectively. The frequency of resistance to other antibiotics was as follow: imipenem (48%), ceftazidime (76%), and amikacin (76.5%). Among the resistant strains, double drug resistance and MDR phenotypes were detected in the frequencies of 10.4% and 66.2%, respectively. All of the metronidazole-resistant strains belonged to *tcdA*^ +^/*tcdB*^ + ^genotype with triple or quintuple drug resistance phenotypes. MIC_50_ and MIC_90_ for this antibiotic was equally ≤ 8 μg/ml.

**Conclusion:**

These results proposed the association of *tcdA*^ +^/*tcdB*^ + ^genotype of *C. difficile* and the emergence of resistance strains to broad-spectrum antibiotics and metronidazole.

## INTRODUCTION

*Clostridium difficile *is an anaerobic, spore-forming, Gram-positive bacterium that is able to colonize the human intestinal tract [1]. Infection with this bacterium can be induced through the consumption of contaminated foods or during hospitalization. The infection shows both colonic and extracolonic symptoms. The colonic infestations vary from asymptomatic state to diarrhea, simple colitis, pseudomembranous colitis, fulminant colitis with perforation, prolonged ileus, megacolon, and death [2, 3]. The extracolonic features include small bowel *C. difficile*-associated diseases (CDAD), bacteremia, and reactive arthritis [4]. The main virulence factors that usually initiate the disease symptoms are two potent toxins, toxin A (enterotoxin) and toxin B (cytotoxin) [5]. 

In most healthy individuals, the growth of *C. difficile* is controlled by the normal microbiota of the intestine, but in disease conditions, the use of antibiotics and medications, such as proton pump inhibitors, possibly cause the bacterium to proliferate [6]. The emergence of resistant strains of *C. difficile* to different antibiotics is now a reason of great concern worldwide [7]. Despite of the evidence on reduced sensitivity of *C. difficile* strains to common therapeutic regimens, the administration of metronidazole is still considered as the best medicine for treatment of the infections caused by this bacterium [8-10]. The effectiveness of this treatment is considerably challenged with the emergence of new epidemic multidrug resistance (MDR) strains in some countries [11]. The MDR strains are matters of serious concern in hospitals that creates an extensive problem in the management of infected patients [11]. 

**Table 1:** The ranges of MIC values and MIC_50_/MIC_90 _results for 86 *C. difficile* isolates

** Antibiotic**	**MIC Ranges** ** (μg/ml)** * **n (%)**					**MIC**_50_ **(μg/ml)**	**MIC**_90_ **(μg/ml)**
Metronidazole	≤8 82 (95%)	16 0 (0%)	32 4 (5%)			≤8	≤8
Amikacin	≤16 17 (20%)	32 3 (3.5%)	64 20 (23%)	≥128 46 (53.5%)		≥128	≥128
Ceftazidime	≤16 21 (24%)	32 10 (12%)	64 20 (23%)	≥128 35 (41%)		64	≥128
Imipenem	≤4 10 (12%)	8 35 (40%)	16 24 (28%)	≥32 17 (20%)		8	≥32
Ciprofloxacin	<4 3 (3%)	4 22 (26%)	8 29 (34%)	16 11 (13%)	≥32 21 (24%)	8	≥32

* The MIC values were determined by agar dilution method using no. 0.5 McFarland standard suspension for each isolate on Brucella agar medium containing 7% defibrinated sheep blood and defined serial two-fold concentrations of each drug.

There is new evidence suggesting the cotransfer of toxin-encoding genes and conjugative transposons encoding resistance to antibiotics among different *C. difficile* strains [12]. This study has shown that three transfer-proficient conjugative transposons in the *C. difficile* genome are close to its pathogenicity locus, which encodes toxins TcdA and TcdB. The epidemiology of *C. difficile*-associated infections, their virulence properties, and antimicrobial resistance will provide new insights to design the best treatment strategies against infections with these strains in different geographic regions. Therefore, in the current study, we aimed to analyze the association between the MDR phenotypes and toxin genotypes of *C. difficile* strains which may infect hospitalized patients under the administrating prophylactic antibiotics for hospital acquired infections. 

## MATERIALS AND METHODS

***Patients and bacterial strains. ***A total of 86 suspicious isolates of *C. difficile *collected from fecal samples of hospitalized patients with intestinal disorders were studied in a referral laboratory at Taleghani Hospital in Tehran, Iran. Bacterial cultivation was carried out on proper culture media (*C. difficile* medium, Mast, United Kingdom) supplemented with 7% horse blood and selective components. The cultured plates were incubated at 37°C for at least 48-72 h under anaerobic conditions (*Anoxomat*, *MART Microbiology*, the Netherlands). The grown colonies were initially characterized based on their colony and cell morphologies and common biochemical test reactions [1]. Further identification of the strains was performed by PCR using specific primers [13]*.*

***DNA extraction and molecular identification. ***DNA extraction from the bacterial strains was carried out using boiling method [13]. For the identification of the suspected colonies, PCR to detect the* cdd3 *gene fragment was amplified by PCR and specific primer pairs Tim6 and Struppi6, as described by Spigaglia *et al.* [14]. To analyze any relationship between the frequency of resistance phenotypes and genotypes of the strains for toxin A and toxin B, *tcdA* and *tcdB* genes were amplified by PCR as described previously [14]. 

***Determination of antibiotic susceptibility patterns and minimal inhibitory concentrations (MIC). ***To analyze the susceptibility of the isolates to metronidazole and common CDAD-associated antibiotics (amikacin, imipenem, ceftazidime, and ciprofloxacin), the standard agar dilution method was used according to the clinical and laboratory standard institute guideline [15]. Fresh colonies of each isolate were suspended in a sterile saline buffer (No. 0.5 McFarland standard), and 20 µl of bacterial suspensions were inoculated onto Brucella agar medium plates (Merck Co, Germany) supplemented with 7% defibrinated sheep blood and defined serial two-fold concentrations of each drug (Table 1). The plates were incubated in an anaerobic jar at 37°C for 24-48 h. MIC of each antibiotic was determined after 48 h of incubation [16]. Cut-off concentrations of ≥32 µg/ml for metronidazole, ≥4 µg/ml for ciprofloxacin, ≥ 16 µg/ml for imipenem, ≥ 64 µg/ml for amikacin, and ≥ 32 µg/ml for ceftazidime were considered as definitive criteria for the detection of the resistant strains (Table 1) [15]. 

***Statistical analysis.*** Chi-square and Fisher’s exact tests were used to analyze the data. A P value less than 0.05 was considered statistically significant.

## RESULTS 

The current study provides a comprehensive analysis of the antibiotic resistance in 86 *C. difficile* clinical strains collected from different hospitals in Tehran (Iran) during a prospective study in 2011. All the suspected isolates were confirmed as *C. difficile *either by conventional or molecular methods. According to the defined *MIC* break points, resistance rates among the 86 strains were 97% for ciprofloxacin, 48% for imipenem, 76.5% for amikacin, 76% for ceftazidime, and 5% for metronidazole. The MIC values for each antibiotic are shown in Table 1. The metronidazole MIC at which 50% (MIC_50_) and 90% (MIC_90_) of the tested isolates were inhibited was equally ≤ 8 μg/ml. Higher MIC_90_ values were found for ciprofloxacin (> 32 μg/ml), imipenem (32 μg/ml), ceftazidime (≥ 128 μg/ml), and amikacin (≥ 128μg/ml). Four isolates (5%) presented elevated MIC for metronidazole (32 μg/ml) whereas MIC of ciprofloxacin was ≥ 4 μg/ml in 97% of the strains, most of them were inhibited by a concentration of ≤ 8 μg/ml of metronidazole. In the case of amikacin and ceftazidime, the prevalence of strains with higher levels of resistance was considerable (53.5% and 41%, respectively). Double resistance to the studied agents was uncommon and was detected in 10.4% of the strains. However, the results showed a higher percentage of MDR phenotype among the *C. difficile* isolates (66.3%). The overall level of multidrug resistance was 36% for the isolates with resistance to at least three drugs (triple drug resistance), 29% for the isolates with resistance to at least four drugs (quadruple drug resistance), and 1.16% for the isolates with resistance to at least five drugs (quintuple drug resistance). Toxinotyping of the MDR *C. difficile* strains for* tcdA* and *tcdB* showed four strains as *tcdA*^ +^/*tcdB* ‾ (7%), one strain as *tcdA*^-^/*tcdB*^ +^ (53%), forty seven strains as *tcdA*^ +^/*tcdB*^ + ^(84.2%), and four strains as *tcdA*‾/*tcdB* ‾ (7%) (Table 2). Concurrent resistance to the tested antibiotics was significantly observed among the strains with *tcd*A^+^/*tcd*B^+^ genotype (*P* = 0.015). All the metronidazole-resistant strains belonged to this genotype group.

**Table 2:** Frequency of multidrug-resistant (MDR) phenotype among 86 *C. difficile* isolates

***tcd*** **A** ^-^ **/B** ^-^	***tcd*** **A** ^+^ **/B** ^+^		***tcd*** **A** ^+^ **/B** ^-^	***tcd*** **A-/B+**		**MDR%** ***	**Frequency n** ** ** (%)**	**MDR phenotype** *
**0**		**1**	**0**		**0**	**1.7%**	**1/86 (1.16%)**	**Quintuple ** **Drug Resistance**
0		1	0		0	1/57 (1.7%)	1/86 (1.16%)	Metronidazole, Ceftazidime, Amikacin, Imipenem, Ciprofloxacin
						
**2**		**20**	**3**		**0**	**44%**	**25/86 (29%)**	**Quadruple Drug Resistance**
2		20	3		0	25/57 (44%)	25/86 (29%)	Ceftazidime, Amikacin, Imipenem, Ciprofloxacin
**2**		**27**	**1**		**1**	**54.2%**	**31/86 (36%)**	**Triple Drug Resistance**
1		12	0		1	14/57 (24.5%)	14/86 (16.2%)	Ceftazidime, Imipenem, Ciprofloxacin
1		8	1		0	10/57 (17.5%)	10/86 (11.6%)	Ceftazidime, Amikacin, Ciprofloxacin
0		2	0		0	2/57 (3.5%)	2/86 (2.32%)	Metronidazole, Ceftazidime, Ciprofloxacin
0		4	0		0	4/57(7%)	4/86 (4.6%)	Amikacin, Imipenem, Ciprofloxacin
0		1	0		0	1/57 (1.7%)	1/86 (1.1%)	Metronidazole, Amikacin, Ciprofloxacin
**1**		**6**	**0**		**2**		**9/86 (10.4%)**	**Double Drug Resistance**
1		2	0		1		4/86 (4.65%)	Amikacin, Ciprofloxacin
0		4	0		0		4/86 (4.6%)	Ceftazidime, Ciprofloxacin
0		0	0		1		1/86 (1.16%)	Ceftazidime, Amikacin

* MDR, strains with triple, quadruple, and quintuple drug-resistant phenotypes were defined as strains with multidrug resistant phenotype to different classes of antimicrobial.

** Frequency of resistance isolates among the total isolated bacteria;

*** Frequency of each resistance group pattern among the isolates with MDR phenotype.

## DISCUSSION

Effective treatment of CDAD is usually based on common sensitivity reports for the strains in each country. There are a few reports about the prevalence of different MDR phenotypes among the clinical isolates in some countries [17, 18]. We report reduced susceptibility of our strains to ciprofloxacin (97%), amikacin (76.5%), and ceftazidime (76%), which were higher than other resistance phenotypes among the studied isolates. Detection of high level fluoro-quinolone-resistant phenotype in *C. difficile* strains was previously reported by Nore´n* et al. *[19] who studied resistance frequency of their isolates to moxifloxacin (23%), levofloxacin (100%), and ciprofloxacin (100%). MIC levels to these antibiotics varied between 0.5 and > 32 mg/L with MIC_50_ of > 32 mg/L in some studies [19, 20]. The estimated MIC levels for ciprofloxacin among the isolates of this study (MIC_50/90_ of 8 and ≥32μg/ml, respectively) proposed lower levels of MIC_50_ among them. The level of resistance to metronidazole varies in different countries. In European countries, MIC_50 _and MIC_90 _for metronidazole varied from 0.25 to 1 µg/ml and 0.5 to 2 µg/ml, respectively [19-22]. The highest reported MIC value for metronidazole is 64 µg/ml that was found in one strain in Hong Kong [23]. 

Data from the present study showed that 95% of our strains were inhibited by metronidazole at a concentration of ≤8 µg/ml; however, 5% of the isolates showed elevated MIC (≥32 µg/ml) that was similar to the overall reported rate of resistance in Spain (6.3%) [24], but higher than results from other studies [19-22]. This resistance level probably was caused by indiscriminate use of metronidazole in CDAD and also in other common cases of protozoal infections in Iran. In the case of ceftazidime, approximately 64% of the isolates showed *in vitro* resistance. In a study conducted in the United States, MIC_90_ of *C. difficile* isolates for ceftazidime were >128 µg/ml [25]. The results of this study showed MIC_50 _and MIC_90 _of 64 and ≥128 µg/ml, respectively. These isolates showed lower resistance rate and MIC value to imipenem (48%, MIC_50/90 _of 8 and 32 µg/ml, respectively) compared with that was determined in Kuwait (86%, with MIC_50/90 _of 32 and > 32 µg/ml, respectively) [26]. 

In this study, the analysis of the drug resistance phenotypes among the isolates showed 17 strains with single drug resistance (19.8%), 9 strains with double drug resistance (10.4%), and 57 isolates with MDR 

phenotypes (66.2%) (Table 2). Triple antibacterial resistance was found as main MDR phenotype among these strains (36%). All the strains with resistance phenotypes to metronidazole belonged to the triple or quintuple drug resistance groups. In a study in Italy, out of 316 *C. difficile* clinical isolates, 12 (3.7%) were resistant to only one antibiotic, 54 (17%) to two antibiotics, and 82 (26%) to at least three antibiotics (MDR) (18), however reduced susceptibility to metronidazole was not found among the MDR strains. 

 In a similar study in Kuwait, while no resistance was detected to metronidazole, MDR phenotype was reported in 55 isolates (75.3%) and double, triple, and quadruple resistance phenotypes were observed in 11%, 38.3%, and 37% of the strains, respectively [26]. Most of the MDR strains in our study were toxigenic (94.2%). Concurrent resistance to the tested antibiotics was significant in the *tcdA*+/*B*+ toxigenic group. These results cast new light into the relationship between toxigenic strains and resistance phenotype in *C. difficile*. This association was previously reported by others [18, 27, 28]. It has been shown that toxigenic strains of *C. difficile* (e.g. NAP1/O27) are resistant to broad spectrum antibiotics, such as beta-lactams, clindamycin, and flouroquinolones [29]. It has been also indicated that mean consumption of several β-lactams, amikacin, imipenem, and fluoroquinolones was higher in affected hospitals with the toxigenic-resistant strains of *C. difficile*, which suggests the involvement of widespread antibiotic prescription in selection of toxigenic strains in these hospitals [30]. The relationship between toxigenicity and resistance phenotype of the *C. difficile* strains was also supported by a recent finding about cotransfer of *C. difficile* pathogenicity locus, encoding the two noted toxins, with conjugative transposons encoding resistance to several antibiotics [12]. *In vitro* transfer of genetic determinants among different strains of *C. difficile* was established by Jorg Wust *et al.* [31] in 1983. They concluded that this transmission cannot occur with plasmid DNA, and mechanism of the transfer seems to be a conjugation-like phenomenon. Pituch *et al.* [32] showed an association between antibiotic resistance strains and toxin B production in Warsaw. Correlation between fluoroquinolone resistance and resistance to macrolide-lincosamide-streptogramin antimicrobials was shown by Ackermann *et al.* [33]. Consistent with these data, our results showed a similar association between the coexistence of *tcdA*+/*tcdB*+ genes and MDR phenotypes among the clinical isolates of *C. difficile*. This finding emphasizes the need for continuous monitoring of antimicrobial susceptibility patterns among the pathogenic strains for prevention of the occurrence of eradication failure in the infected patients. 
